# ADSCs-derived exosomes ameliorate hepatic fibrosis by suppressing stellate cell activation and remodeling hepatocellular glutamine synthetase-mediated glutamine and ammonia homeostasis

**DOI:** 10.1186/s13287-022-03049-x

**Published:** 2022-10-04

**Authors:** Baitong Wu, Jiuxing Feng, Jingyi Guo, Jian Wang, Guanghui Xiu, Jiaqi Xu, Ke Ning, Bin Ling, Qingchun Fu, Jun Xu

**Affiliations:** 1 East Hospital, School of Medicine, Tongji University, Shanghai, 200120 People’s Republic of China; 2 Key Laboratory of Medical Epigenetics and Metabolism, Institutes of Biomedical Sciences, Fudan University, Shanghai, People’s Republic of China; 3 Department of Intensive Care Unit, Affiliated Hospital of Yunnan University (The Second People’s Hospital of Yunnan Province), Yunnan University, Kunming, People’s Republic of China; 4 Department of Neuroscience, Sheffield Institute for Translational Neuroscience, University of Sheffield, Sheffield, UK; 5 Shanghai Public Health Clinical Center, Fudan University, Shanghai, People’s Republic of China

**Keywords:** Adipose-derived stromal cells, Exosomes, Hepatic fibrosis, Glutamine synthetase

## Abstract

**Background:**

Hepatic fibrosis is a common pathologic stage in chronic liver disease development, which might ultimately lead to liver cirrhosis. Accumulating evidence suggests that adipose-derived stromal cells (ADSCs)-based therapies show excellent therapeutic potential in liver injury disease owing to its superior properties, including tissue repair ability and immunomodulation effect. However, cell-based therapy still limits to several problems, such as engraftment efficiency and immunoreaction, which impede the ADSCs-based therapeutics development. So, ADSCs-derived extracellular vesicles (EVs), especially for exosomes (ADSC-EXO), emerge as a promise cell-free therapeutics to ameliorate liver fibrosis. The effect and underlying mechanisms of ADSC-EXO in liver fibrosis remains blurred.

**Methods:**

Hepatic fibrosis murine model was established by intraperitoneal sequential injecting the diethylnitrosamine (DEN) for two weeks and then carbon tetrachloride (CCl_4_) for six weeks. Subsequently, hepatic fibrosis mice were administrated with ADSC-EXO (10 μg/g) or PBS through tail vein infusion for three times in two weeks. To evaluate the anti-fibrotic capacity of ADSC-EXO, we detected liver morphology by histopathological examination, ECM deposition by serology test and Sirius Red staining, profibrogenic markers by qRT-PCR assay. LX-2 cells treated with TGF-β (10 ng/ml) for 12 h were conducted for evaluating ADSC-EXO effect on activated hepatic stellate cells (HSCs). RNA-seq was performed for further analysis of the underlying regulatory mechanisms of ADSC-EXO in liver fibrosis.

**Results:**

In this study, we obtained isolated ADSCs, collected and separated ADSCs-derived exosomes. We found that ADSC-EXO treatment could efficiently ameliorate DEN/CCl_4_-induced hepatic fibrosis by improving mice liver function and lessening hepatic ECM deposition. Moreover, ADSC-EXO intervention could reverse profibrogenic phenotypes both in vivo and in vitro, including HSCs activation depressed and profibrogenic markers inhibition. Additionally, RNA-seq analysis further determined that decreased glutamine synthetase (Glul) of perivenous hepatocytes in hepatic fibrosis mice could be dramatically up-regulated by ADSC-EXO treatment; meanwhile, glutamine and ammonia metabolism-associated key enzyme OAT was up-regulated and GLS2 was down-regulated by ADSC-EXO treatment in mice liver. In addition, glutamine synthetase inhibitor would erase ADSC-EXO therapeutic effect on hepatic fibrosis.

**Conclusions:**

These findings demonstrated that ADSC-derived exosomes could efficiently alleviate hepatic fibrosis by suppressing HSCs activation and remodeling glutamine and ammonia metabolism mediated by hepatocellular glutamine synthetase, which might be a novel and promising anti-fibrotic therapeutics for hepatic fibrosis disease.

**Supplementary Information:**

The online version contains supplementary material available at 10.1186/s13287-022-03049-x.

## Background

Hepatic fibrosis, as a major part of chronic liver disease, was defined as a scarring response of constantly wound-healing process in liver, which was caused by a variety of reasons, like virus infection, drug abuse, immune dysregulation, alcohol or metabolic disorder [1, 2]. Tens of millions people worldwide were suffering for this dramatically expanding disease, which may rapidly deteriorate into liver cirrhosis, ultimately leading to liver failure even death [3]. Additionally, almost all types of liver disease morbidity, including cirrhosis [4] and hepatocellular carcinoma (HCC) [5], were with highly correlated with progressive fibrosis. However, such a global public health problem was neglected for a long period. Hepatic fibrosis was not only characterized by excessive extracellular matrix (ECM) deposition and myofibroblasts-derived hepatic stellate cells (HSCs) activation [6–8], but also involving in hepatocytes damage and fibrotic niche formation [9]. Thus, the anti-fibrotic strategies were mainly carried out from three dimensions: (a) dampen the fibrogenic activation of myofibroblasts [10]; (b) inhibit apoptosis and necroptosis of liver functional cells [11]; and (c) improve the liver tissue microenvironment and liver fibrosis such as inflammation modulation [12]. Although some inhibitors targeting to monocytes infiltration had been shown potential effects on hepatic fibrosis, there was not any approved drugs for clinical application. Therefore, drugs for clinical treatment of hepatic fibrosis needed to be further explored and developed.

In recent years, researchers found that mesenchymal stem cells (MSCs)-based therapy exhibited multiple therapeutic effects in liver diseases, which included functional cells maintenance, immunology modulation and tissue repair [13–15]. According to clinical trial database (https://www.clinicaltrials.gov/), it showed that more than 60 MSCs-based clinical trials had been implemented for liver diseases, and 11 of which had been completed [16]. Various source of MSCs like bone marrow-derived MSCs, umbilical cord-derived MSCs and adipose tissue-derived MSCs fully showed therapeutics effectiveness and safety for liver pathologies [17–19]. Particularly, adipose-derived stromal cells (ADSC), as an easier accessible and abundant type of MSCs, had gradually become a major tool in this area, which was at the serve of tissue engineering and regenerative medicine. Except for the basic MSCs properties, ADSCs possessed strong capability of proliferation and expansion in comparison with BM-MSCs [20], and also paracrine and immunomodulatory properties related to its specific secretome and the soluble factors within it [21]. Our research team had previously shown the therapeutic potential of ADSCs in motor neuron disease [22, 23], tissue injury disease [24, 25] and stroke syndromes [26]. In spite of these advantages, cell-based therapy transplantation still limited to cell storage, cells availability, cell engraftment efficacy and other immunoreaction problems. Just at this time, a growing number of studies emerged and suggested that MSCs could promote tissue repair through paracrine way, depending on these secreted bioactive factors [27]. This novel viewpoint prompted the development of MSCs derivate.

Exosomes (EXO) were spherical membrane vesicles with double-lipid layer structure, its diameter ranging from 40 to 150 nm, which were of vital importance in immune regulation and intercellular communication [28]. Different from another major type of EVs-ectosomes, the biogenesis of exosomes was of endosomal origin, which involved the process of sequential plasma membrane invagination and intracellular multivesicular bodies (MVBs) formation [29]. The generated exosomes contained diverse biological molecular (including nucleic acids, cytokines, growth factors, microRNAs, lipids), which might act as a natural high-performance cell-free delivery system. Recently, researchers confirmed that ADSCs-derived exosomes (ADSC-EXO) could ameliorate acute liver injury (ALI) via exosomal cargo miR-17, reducing systematic inflammation [30]. Also, ADSC-EXO treatment in ALI exhibited anti-oxidative stress ability by down-regulated NADPH oxidase 1 (NOX1) and NOX2 [31]. In our previous study, ADSC-EXO also showed significant anti-apoptotic and anti-inflammatory effects in acute liver failure rats, meanwhile reducing hepatocyte necrosis and inflammatory cell infiltration [32]. However, there was indistinct evidence to confirm whether ADSC-EXO could attenuate hepatic fibrosis and its related molecular mechanism.

In this study, we aimed to explore the therapeutic effect of ADSC-EXO on one of the typical chronic liver diseases—hepatic fibrosis. Firstly, isolated human ADSCs and collected ADSC-EVs were characterized. Then, we established DEN/CCl_4_-induced hepatic fibrosis mice model, and partial hepatic fibrosis mice were received ADSC-EXO treatment for 2 weeks through tail vein injection. The ADSC-EXO therapeutic effects on hepatic fibrosis of liver pathology, hepatic biological function and fibrotic phenotypes were evaluated. To confirm the ADSC-EXO effect on activated HSCs, LX-2 cells treated with TGF-β (10 ng/ml) for 12 h were co-incubated with ADSC-EXO for 24 h for further analysis. Afterward, to elucidate the underlying mechanism of ADSC-EXO administration, RNA-sequencing analysis (RNA-Seq) was performed in liver tissue of hepatic fibrosis mice and hepatic fibrosis mice treated with ADSC-EXO. Then, we discovered that hepatic glutamine synthetase (GLUL) as a key enzyme in glutamine metabolism was possibly regulated by ADSC-EXO treatment.

## Methods

### Isolation and identification of ADSCs

Adipose tissue was collected by liposuction from healthy donors. The process and protocols were approved by the People's Liberation Army No. 85 Hospital, Shanghai, P.R. China (review serial number NO.2013/18). All donors signed informed consent. After washed with phosphate-buffered solution (PBS) (Gibco, USA), 500 mg adipose tissue about 1 mm^3^ pieces was digested by 0.1% collagenase I (Gibco, USA) for 45 min. Then, we added equal volume of complete culture medium (DMEM-F12 culture medium (Gibco, USA), 10% FBS, 1% Penicillin–Streptomycin) and centrifuged for 10 min in 1000 rpm. After resuspended with PBS and filtered with 40 μm cell strainer, cells were seeded at a density of 1 × 10^6^/ml in complete culture medium containing10ng/ml bFGF (Stem Cell, USA). When the adherent cells approached 80%-90% confluence, cells should be passaged in 1:3.

ADSCs surface markers identification was detected by flow cytometric method with flow cytometer (FACSCalibur, BD, USA). 500 μl ADSCs (3rd passage) suspension was collected and loosed in single-cell status, and then, according to the experiment design anti-human CD44-PE, CD90-FITC, CD73-APC, CD105-PerCP-cy5.5 monoclonal antibodies (eBioscience, USA) were incubated with cells for 30 min at 4 °C, respectively, and washed for detection. Data were analyzed with Flow Jo V10 software.3

Cytomorphology of ADSCs was observed under optic microscope.

### Separation and characterization of ADSC-EXO

To obtain the ADSCs-derived exosomes, when 2nd passage approached 80–90% confluence, the DMEM-F12 culture medium containing 10% EVs-depleted serum and 10 ng/ml bFGF were changed for 24 h and then the cell supernatant was collected. After filtered with 0.22 μm filter, the cell supernatant was pre-centrifuged in 500 g for 10 min, 2000 g for 20 min to remove cells or apoptotic bodies and 10000 g for 60 min to remove cell debris. Then, all supernatant was transferred to Ultracentrifuge tube (Beckman, #355618) and was ultracentrifuged in 120000 g for 70 min to separate exosomes. Then, the pellets were resuspended by 100–150 μl PBS per tube. All procedures were all carried out at 4 °C and obtained exosomes be stored in − 80 ℃ or used immediately.

For exosomes concentration, particle size distribution and purity, the Nanoparticle Tracking Analysis (NTA) was determined by (NanoSight300, Malvern Instruments Ltd, UK). Samples were diluted with 1 ml PBS and prepared as optimal concentration and then loaded into sample cubicle. Then, these particles were tracked and illuminated by laser light under Brownian motion condition. Each sample was repeated and recorded this process for 3 times. The concentration, size distribution and scatter intensity of particles were obtained through Stokes–Einstein equation calculation.

For ultrastructure identification, the exosomes were observed by transmission electron microscopic (TEM). Images of ADSC-EXO were taken by using Hitachi HT7800 electron microscope (HT-7800, Hitachi, Japan).

### Liver fibrosis mouse model and grouping

C57/BL6 mice were purchased from Shanghai SLAC Laboratory Animal Co. Ltd. (Shanghai, China). Animals experiment were approved by Ethical Committee of Laboratory Animals Research Center, Tongji University. The approval No. was TJAA07620401. To establish liver fibrosis mouse model, 2-week-old male mice were injected diethylnitrosamine (DEN, Sigma-Aldrich,20 mg/kg) once a week for 2 weeks and then injected CCl_4_ (5 ml/kg) 3 times per week for 6 weeks, with only oil serving as vehicle group.

Additionally, for ADSC-EXO treatment group, the mice would be administrated ADSC-EXO 10 μg/g mouse through tail vein injection for three times in 2 weeks; for DEN/CCl_4_ + EXO + MSO group, the mice would be administrated MSO (methionine sulfoximine, 10 mg/kg, *i.p*, Sigma-Aldrich, USA*.*) just after ADSC-EXO treatment for three times in 2 weeks, which was an inhibitor of glutamine synthetase.

Finally, all mice were killed. Mice serum and plasma was separated by centrifuging the whole blood (4000 rpm, 15 min, room temperature) for hepatic function test, and liver tissue was harvested for pathological analysis and further analysis.

### Cell lines and cell culture

Immortalized human hepatic stellate cell line LX-2 was purchased from the Chinese Academy of Sciences (Shanghai, China) and cultured in DMEM high-glucose culture media (Gibco, USA) with 10% fetal bovine serum (FBS) (Gibco, USA) and Penicillin–Streptomycin (100U/ml, 100 mg/ml) (Gibco, USA). For LX-2 activation, cultured LX-2 was starved in 1% FBS culture medium for 24 h and then stimulated with human transforming growth factor (TGF-β1, 10 ng/mL; Novoprotein, China) for 12 h. In ADSC-EXO treatment group, the activated LX-2 cells were exposed in ADSC-EXO (200 μg/ml) for 24 h.

Mouse hepatocyte cell line AML12 was purchased from the Chinese Academy of Sciences (Shanghai, China) and cultured in AML12 specific culture medium (Procell, Wuhan, China). Mouse hepatic stellate cell (CP-M041) was purchased from Procell (Wuhan, China) and maintained in specific hepatic stellate cell medium (CM-M041, Procell, Wuhan, China). For mouse hepatic stellate cell activation, it was treated with mouse TGF-β1 (10 ng/mL; Novoprotein, China) for 12 h. To mimic the hepatocytes injury of hepatic fibrosis, AML12 cells were treated with TGF-β1 (5 ng/ml) for 48 h. In ADSC-EXO treatment group, the injured hepatocytes were then exposed in ADSC-EXO (200 μg/mL) for 24 h. Then, the conditional medium of AML12 cells was collected and transferred to activated mouse HSC cells for 24 h to evaluate the crosstalk between HSCs and hepatocytes.

Human ADSCs were primary cells isolated from adipose tissue, and they were established and cultured as described above.

All cells were incubated in 37 °C and 5% CO_2_ incubator (Thermo, USA).

### Western blots

Total protein of liver tissue or cells was lysed by RIPA buffer (1 × , containing 10 mM PMSF protease inhibitor) for 30 min on ice. The protein concentration was quantified with BCA kit (Beyotime Biotech, Shanghai, China). Then, the sample with protein loading buffer (5 × , Beyotime) was performed with the SDS-PAGE and the gels were transferred to PVDF membranes (Millipore, USA). After blocking non-specific signal, the protein bands were incubated with primary antibodies in 4℃ overnights. Following antibodies were used: Alix (92880S, CST, USA), Tsg101(A2216, ABclonal, China), CD63 (Santa Cruz, sc5275, USA), Calnexin (A4846, ABclonal, China), α-SMA (ab124964, Abcam, USA), Collagen I (abs131984, Absin, China), glutamine synthetase (Glul) (A19641, ABclonal, China), Lamin B (66095-1-Ig, Proteintech, China) and GAPDH (2118S, CST, USA). Afterward, secondary antibodies were incubated at room temperature for 1 h and bands were visualized with ECL kit (Abcam, USA) by chemical image system (Amersham Imager 600, USA).

### RNA extraction and quantitative reverse transcription PCR (qRT-PCR)

Total RNA of liver tissues and LX-2 cells was isolated with Trizol reagent (Invitrogen) according to the manufacturer's instructions. Qualified RNA was reverse transcribed into cDNA using EasyScript® One-Step gDNA Removal and cDNA Synthesis SuperMix (TransGene Biotech, China). qRT-PCR assay was performed using ChamQ SYBR qPCR Master Mix (Vazyme, China) in LightCycler96 real-time PCR system (Roche, USA). β-actin served as internal control. The 2-ΔΔCt method was used for data analysis. All primers were synthesized by HuaGene Company (Shanghai, China), and the sequences are listed in Additional file 1: Table S1.

### Hepatic function test

Full-automatic chemistry analyzer (Rayto, China) was used for the hepatic function test. ALT and AST level in mice serum was examined.

### Hematoxylin and eosin (H&E), immunohistochemical (IHC) and Sirius Red staining

Liver tissues in each group were fixed in formaldehyde over 48 h, then embedded in paraffin and forward sliced into 5-μm-thick sections. According to the standard procedures, liver tissues slices were subjected to H&E staining and Sirius Red staining.

For IHC staining of α-SMA and Collagen I, mice liver tissues slice staining was performed as standard protocol described. During the assay, anti-α-SMA (Abcam, USA) and anti-Collagen I (Absin, China) were used as primary antibody and HRP-conjugated goat anti-rabbit IgG was used as the secondary antibody and 3,3′-Diaminobenzidine (DAB) used as the chromogen (ZSGO-BIO, China).

For immunofluorescence staining, paraffin-embedded liver tissues slice was proceeded with standard protocols and then co-stained with primary antibodies anti-mouse ALB antibody (1:500, NB600-41,532, Novus Biologicals, USA) and anti-Glul antibody (1:500, ABclonal, China) and incubated with fluorescence secondary antibody, 4′,6-diamidino-2-phenylindole (DAPI) for nucleus staining.

All images were captured by upright fluorescence microscope OLYMPUS BX53.

### ELISA assay

Collagen III (COLIII) and hyaluronic acid (HA) concentration in mice serum was detected using COLIII (#SU-B29885, RuiXin-Bio, China) and HA (#SU-B20667, RuiXin-Bio, China) ELISA kit. All procedures were followed the manufacture instructions.

### CCK8 assay

Cell proliferation ability of LX-2 cells in different treatment group was evaluated by cell counting kit-8 (CCK8; Beyotime, China). LX-2 cells were pre-seeded in 96-well plate (5000 cells/well) and cultured in cell incubator overnight. 10 μl CCK8 agent was added into LX-2 cell with given different treatments per well (3 replicate wells). For 0 h, 2 h, 4 h, 6 h treatment, the OD450 values of cells were detected by microplate reader (SpectraMax M5, Molecular Devices, USA).

### RNA-sequencing

Liver samples of Control (DEN/CCl_4_) group and ADSC-EXO (DEN/CCl_4_ + ADSC-EXO) treatment group mice were collected (n = 4/group). Then, the total RNA was extracted as above. The quality of RNA was validated using the Agilent 2100 Bioanalyzer (Agilent Technologies, USA). Then, the cDNA libraries were sequenced on Illumina Genome Analyzer platform (Shanghai Personal Biotechnology Co. Ltd, China). Differentially expressed genes (DEGs), Go enrichment and KEGG analysis were conducted on Geneclouds Platform (Shanghai Personal Biotechnology Co. Ltd, China).

### Plasma ammonia determination

Ammonia level in mice plasma was determined using commercial Ammonia Assay kit (Abcam, ab83360). All procedures were according to the manufacturer’s instruction.

### Glutamate and glutamine assay

Glutamate and glutamine level in mice liver tissue was assessed measured using commercial Glutamate Assay kit (Abcam, ab138883) and Glutamine Assay kit (Abcam, ab197011), respectively. All procedures were according to the manufacturer’s protocols.

### Glutathione (GSH) Measurement

The GSH level in mice liver tissue was measured using a Glutathione Assay Kit (Abcam, ab235670). All procedures were according to the manufacturer’s protocols.

### Ethics statement

Animals experiment were approved by Ethical Committee of Laboratory Animals Research Center, Tongji University. The approval No. was TJAA07620401. Adipose tissue for experiments was collected by liposuction operation from young healthy donors, which had approved by the ethical committee of the People's Liberation Army No. 85 Hospital, Shanghai, P.R. China (review serial number NO.2013/18). All participants signed informed consent. All operation was conducted in accordance with the regular procedures and Declaration of Helsinki.

### Statistical data and analysis

Data were analyzed with GraphPad Prism version 7.0 Software. Quantitative data were shown as means ± standard deviation (${\overline{\chi }}$ ± SEM), and T test and one-way ANOVA were used for intergroup and multigroup difference analysis. *P* < 0.05 was considered as significantly different.

## Results

### ADSCs isolation and ADSCs-derived exosomes characterization

**Fig. 1:**
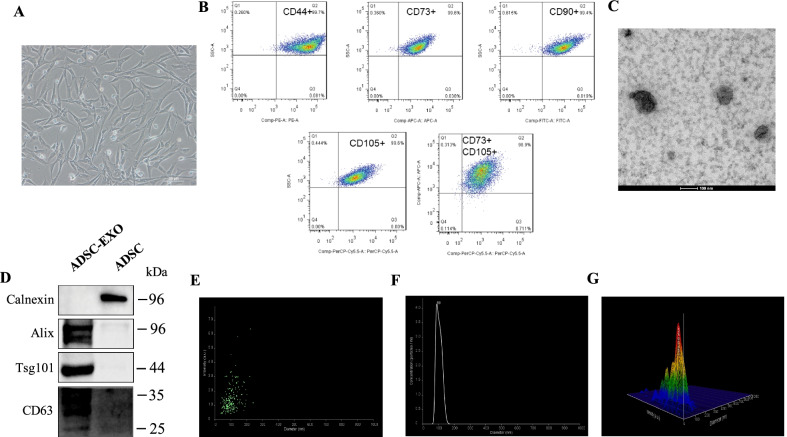
Identification of ADSCs and ADSCs-derived exosomes. **A** Cytomorphology of P3-ADSCs was observed under optic microscope. **B** Flow cytometry analysis for ADSCs markers (including CD44, CD73, CD90, CD105) in P3-ADSCs. **C** Transmission electron microscopic (TEM) analysis for ADSCs-derived exosomes (scale bar 100 nm). **D** Western blot analysis of exosomal protein markers Alix, Tsg101, CD63 and ER marker Calnexin in ADSC-EXO lysate, ADSC protein as control. **E–G** Nanoparticle tracking analysis (NTA) for ADSC-EXO size distribution, concentration and intensity

The isolated human ADSCs were cultured as previously reportedly [24]. As shown in Fig. 1A, under the electronic microscope ADSCs of passage 3 spread as fibroblast-like appearance and proliferate with uniform size and density in a swirling adherent way. Then, the isolated ADSCs were identified with the mesenchymal stem cell markers (Fig. 1B). Flow cytometry analysis indicated that ADSCs revealed high expression of mesenchymal stem cells markers, including 99.4% of CD90^+^, 99.7% of CD44^+^, 99.6% of CD105^+^, 99.6% of CD73^+^ (98.9% of CD73^+^–CD105^+^). Besides, in our previous studies, we have identified the multipotency of isolated ADSCs with tri-lineage differentiation assay, including adipogenesis, chondrogenesis and osteogenesis [21–23]. These results indicated qualified ADSCs were obtained. Then ADSC-derived exosomes were collected and separated as previously descried [30]. We isolated ADSC-EXO using ultracentrifuge method. TEM analysis showed that isolated ADSC-EVs showed ultrastructure of spherical or saucer shape (Fig. 1C). Exosomes associated protein markers were also expressed in isolated ADSC-EVs, including Alix, Tsg101 and CD63; meanwhile, endoplasmic reticulum (ER) membrane marker Calnexin was not expressed but in ADSCs cells (Fig. 1D). In addition, Nanoparticle Tracking Analysis (NTA) was conducted for further exosomes characteristics identification. Individual particles were tracked the Brownian motion and then concentration, size distribution and scatter intensity of particles were obtained through Stokes–Einstein equation calculation. Diameter–concentration curve showed that ADSCs-EVs diameter ranged from 30 to 150 nm with a mean of 89 nm (Fig. 1F), which was accord with exosomes size distribution, and the mean concentration was 4.5 × 10^7^ particles/ml. The diameter–intensity curve also showed that the intensity of ADSCs-EVs was in an interval of 0.5–6.3 a.u. (Fig. 1E) and reached to the highest level when the particles dimension was about 150 nm (Fig. 1G). As shown in Fig. 1C–G, we characterized the isolated ADSCs-EVs and validated it to meet the requirements [28] from exosomal morphology, size distribution and concentration, protein markers, etc. Above all, these results indicated that we successfully isolated ADSCs, collected and separated ADSCs-derived exosomes.

### ADSC-derived exosomes treatment ameliorates DEN/CCl_4_-induced hepatic fibrosis

**Fig. 2:**
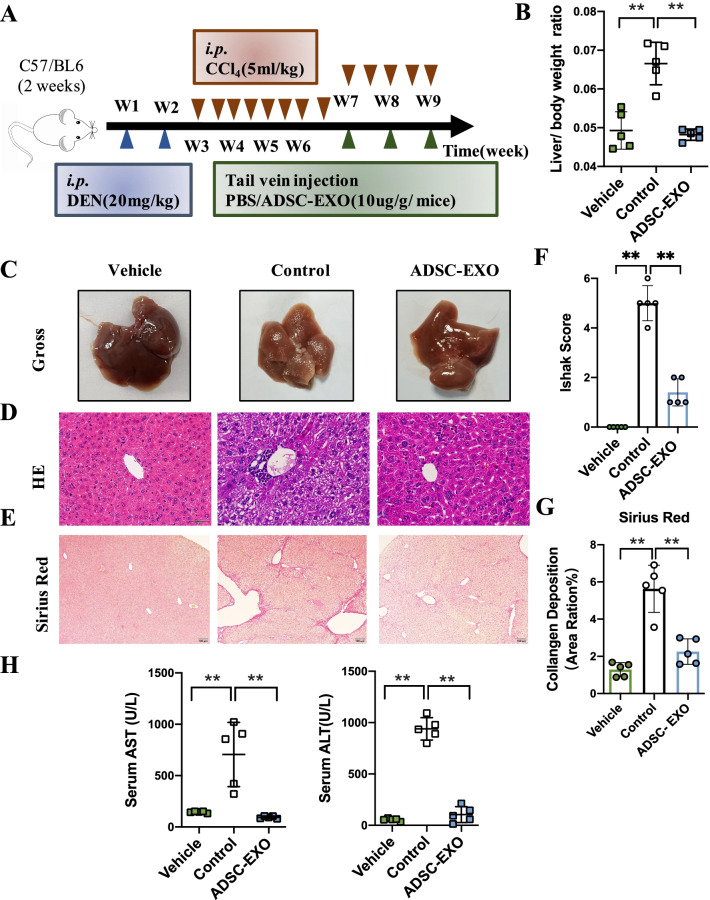
ADSC-EXO treatment ameliorates hepatic function in DEN/CCl_4_-induced liver fibrosis mice. **A** Schematic diagram of DEN/CCl_4_-induced liver fibrosis and ADSC-EXO treatments strategy in mice. **B** Liver/body weight ratio of vehicle (PBS), control (DEN/CCl_4_) and ADSC-EXO (DEN/CCl_4_ + ADSC-EXO) mice (n = 5/group). **C** Representative images of liver gross. **D** H&E staining of liver morphology in each group mice (scale bar, 50 μm). **E** Sirius Red staining of collagen deposition (scale bar, 100 μm) in each group mice. **F** Ishak score statistical of each group mice. **G** Quantitative statistics of Sirius Red staining by Image J software analysis. **H** Hepatic function serology markers AST and ALT level of mice serum at mice killed timepoint. All data are shown as the mean ± SEM, *p < 0.05, **p < 0.01 comparing with corresponding controls by unpaired t test between two groups

According to the basis of preliminary work, ADSC-EXO have shown remarkable therapeutic potential in a variety of tissue injury diseases, including osteoarthritis, amyotrophic lateral sclerosis (ALS), diabetic foot, etc. Hepatic fibrosis, as an abnormal proliferation of connective tissue in liver, is a reversible pathophysiological process in early development. We sought to figure out whether ADSC-EXO could improve hepatic fibrosis. To determine this, we generated a hepatic fibrosis mice model by continually intraperitoneal injection of DEN/CCl_4_ with fixed frequency as shown in Fig. 2A. ADSC-EXO treatment group mice were infused in a dose of 10 μg/g mice ADSC-EXO through tail vein injection for 3 times in 2 weeks. Mice body weight and liver weight were measured at killed point. We found that liver/body weight ratio was significantly elevated in control group than vehicle group, which indicated severe liver fibrosis process. But ADSC-EXO treatment significantly decreased liver/body weight ratio compared with control group (Fig. 2B). Gross examination showed that the mice hepatic edge of control group was obviously round and blunt, and the capsule was tight and hard in texture, with an uneven and granular surface (Fig. 2C). However, in ADSC-EXO treatment mice, the liver tissue was soft in texture, with more smoothly surface, recovered elasticity, the capsule relaxed and the hepatic granular disappeared. Besides, liver fibrosis degree was assessed by H&E staining and Sirius Red staining. H&E staining results suggested that the inflammatory infiltrated cells in ADSC-EXO treatment mice were less than that in untreated control group (Fig. 2D). In Sirius Red staining, liver sections of control mice showed severe collagen deposition, but dramatically remission in ADSC-EXO treatment mice (Fig. 2E). The quantification of Sirius Red staining is shown in Fig. 2G. Moreover, by integrating two aspects of results, we evaluated ADSC-EXO diagnosis effect on hepatic fibrosis of mice using Ishak score system (Fig. 2F). It showed that scores in control group all ≥ 4, while with ADSC-EXO treatment all ≤ 2. Besides, hepatic functional markers of alanine aminotransferase (ALT) and aspartate aminotransferase (AST) were observed significant elevation in control mice serum but nearly return to normal level in ADSC-EXO group (Fig. 2H). Collectively, these data suggested that ADSC-EXO intervention could ameliorate DEN/CCl_4_-induced hepatic fibrosis.

### ADSC-EXO treatment reverses profibrogenic phenotypes in DEN/CCl_4_-induced hepatic fibrosis mice

**Fig. 3:**
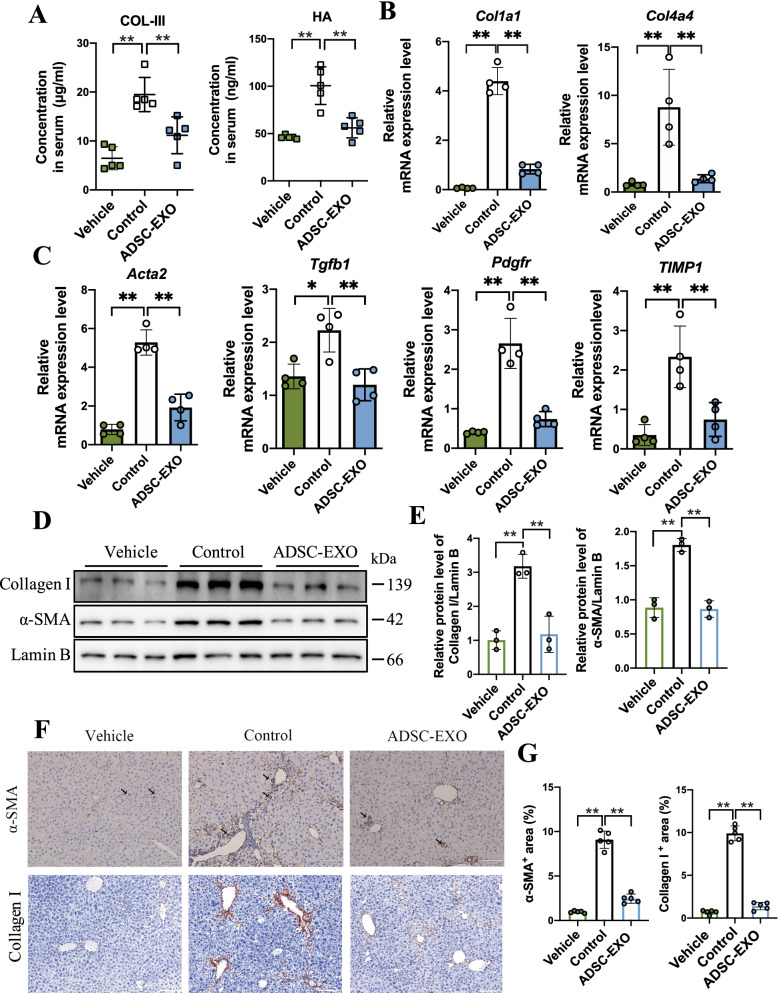
ADSC-EXO reverses profibrogenic phenotypes in CCl_4_/DEN-induced liver fibrosis mice. **A** Collagen III (COL-III) and hyaluronic acid (HA) concentration in mice serum was measured by ELISA assay (n = 5/group). **B** Relative mRNA expression level of collagen deposition marker Collagen I and Collagen VI encoding gene *Col1a1* and *Col4a4* in mice liver tissue was determined by qRT-PCR analysis (4 replicates). **C.** Relative mRNA expression level of pro-fibronectin genes *Acta2, Tgfb, Pdgfr, TIMP1* in mice liver tissue was determined by qRT-PCR analysis (4 replicates). **D** Protein expression level of α-SMA and Collagen I in mice liver tissue was detected by western blot analysis. (Each lane represents one mouse, 3 replicates.) GAPDH as internal reference. **E** Quantification statistic of (D). **F** Representative immunohistochemical staining images (IHC) of α-SMA in mice liver slices (scale bar, 100um; black arrows represent α-SMA positive area). **G** Quantification statistic of α-SMA^+^ area% in α-SMA IHC slices by Image J software analysis (5 replicates). **H** Representative IHC images of Collagen I in mice liver slices (scale bar, 100um; black arrows represent Collagen I positive area). **I** Quantification statistic of Collagen I ^+^ area% in Collagen I IHC slices by Image J software analysis (5 replicates). All data are shown as the mean ± SEM, *p < 0.05, **p < 0.01 comparing with corresponding controls by unpaired t test between two groups

Based on these preliminary findings, we next evaluated if the ADSC-EXO played a functional role in DEN/CCl_4_-induced liver fibrosis mice. As well known, collagen deposition in fibrotic liver tissue was a typical phenotype. First, we measured Collagen III and HA concentration in mice serum at killed point by ELISA assay, which was a golden standard to reflect fibrosis level. As expected, the serum Collagen III and HA concentration of mice administrated ADSC-EXO was decreased compared with control group (Fig. 3A). Then, we assessed the mRNA levels of collagen deposition markers *Col1a1* and *Col4a4* in mice liver tissue using qRT-PCR analysis, which showed that the *Col1a1* and *Col4a4* mRNA level was reduced substantially in ADSC-EXO group (Fig. 3B). Next, we examined mRNA expression level of classical fibrogenic gene involving in fibrogenesis process, including *Acta2*, *Pdgfr*, *Tgfb1*, *TIMP1*. qRT-PCR analysis revealed that the mRNA level of these genes was down-regulated after ADSC-EXO treatment (Fig. 3C).

In hepatic fibrosis process, hepatic stellate cells (HSCs) trans-differentiation or activation leads to matrix protein secreting, forming fibrous scar afterward, and developing to in liver fibrogenesis [8]. Thus, we tested the effect of ADSC-EXO on HSCs activation by anti-α-SMA IHC staining in mice liver sections, and we found that the α-SMA-positive cells surrounding portal vein and central vein (cv) significantly reduced in ADSC-EXO mice compared to control mice (Fig. 3F–G). Another HSCs marker Collagen I also displayed similar results (Fig. 3F, G). Note that western blot analysis further supported this result, suggesting that the HSC activation was suppressed by ADSC-EXO intervention (Fig. 3D–E).

These data confirmed that ADSC-EXO could reverse the profibrogenic phenotypes of hepatic fibrosis from multiple aspects, mainly focusing on collagen deposition reduction, activated HSCs abrogation and profibrogenic signaling inhibition.

### ADSC-EXO suppresses HSCs activation and proliferation in vitro

**Fig. 4:**
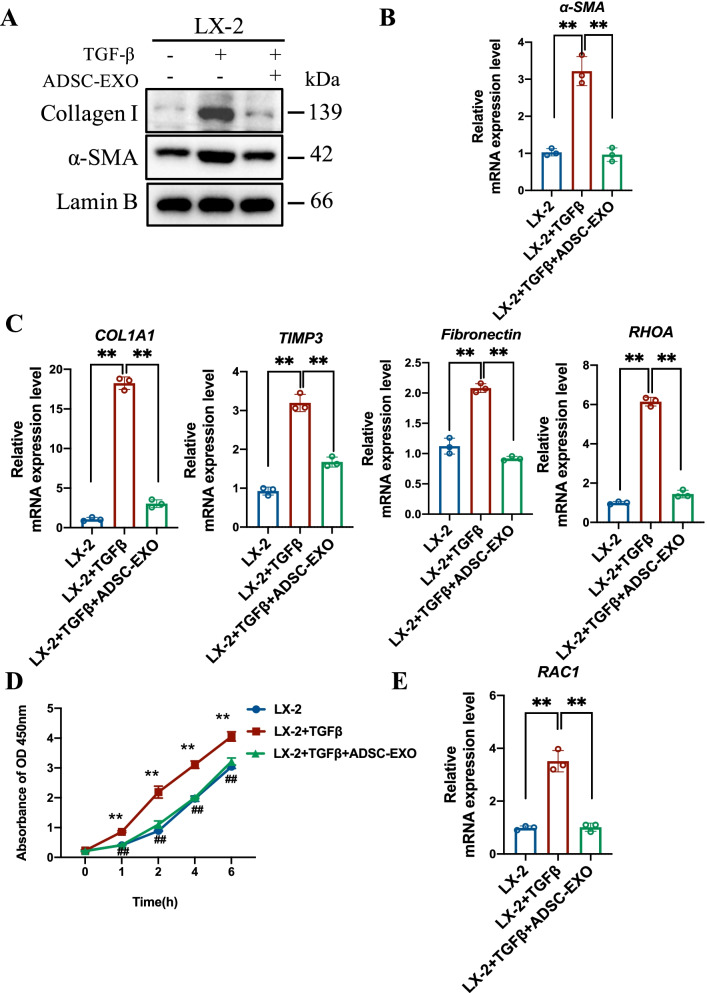
ADSC-EXO treatment suppresses HSCs activation and attenuates its profibrogenic phenotype in vitro. **A** Western blot analysis of HSCs markers α-SMA and Collagen I protein level in untreated LX-2 cells, LX-2 cells with 12 h TGF-β treatment (10 ng/mL) for activation and activated LX-2 cells exposed to ADSC-EXO (200 μg/ml) for 24 h. GAPDH as internal reference. **B** Relative mRNA expression level of α-SMA in LX-2 cells with different treatments was determined by qRT-PCR analysis (3 replicates). **C** Relative mRNA expression level of profibrogenic-associated genes *COL1A1, TIMP3, Fibronectin, RHOA* in LX-2 cells was determined by qRT-PCR analysis (3 replicates). **D** CCK8 assay was performed for LX-2 cells proliferation ability measurement in each group. 1 h, 2 h, 4 h and 6 h represent ADSC-EXO exposure duration (3 replicates). **E** Relative mRNA expression level of cell proliferation marker RAC1 in LX-2 cells was determined by qRT-PCR analysis (3 replicates). All data are shown as the mean ± SEM, *p < 0.05, **p  < 0.01 comparing with corresponding controls by unpaired t test between two groups

In DEN/CCl_4_-induced hepatic fibrosis mice model, we found that activated HSCs in ADSC-EXO treatment group were significantly decreased. Accordingly, to further explore the potential function of ADSC-EXO on HSCs in vitro, we generated TGF-β-induced LX-2 cell activation model. After TGFβ (10 ng/mL) induction for 12 h, western blot results showed α-SMA and Collagen I expression level in TGFβ-treated LX-2 cells was significantly higher than untreated cells (Fig. 4A). The mRNA level of α-SMA in LX-2 cells was also in line with the protein expression level, which both indicated that LX-2 cells have turned from quiescent HSCs into activated myofibroblast-like cells (Fig. 4A–B). However, the α-SMA mRNA level and protein level were both down-regulated with 24 h ADSC-EXO treatment in activated LX-2 cells (Fig. 4A–B). Subsequently, we also evaluate the mRNA level of some key profibrogenic genes using qRT-PCR analysis. The results revealed that 2 classical collagens-associated genes COL1A1 and tissue inhibitor metalloproteinases 3 (TIMP3) of activated LX-2 cells showed markedly decrease in mRNA level after ADSC-EXO treatment (Fig. 4C). Usually, COL1A1 is responsible for collagen production, also as Collagen I encode gene, and TIMP3 for extracellular matrix (ECM) deposition. TIMP3, as an important endogenous inhibitor of matrix metalloproteinases (MMPs), plays a decisive role in ECM integrity, which could inhibit the natural decomposition process of collagens by MMPs [33]. In addition, compared with activated LX-2 cells, *Fibronectin* and *RhoA* mRNA level also exhibited dramatically reduction in activated HSCs with ADSC-EXO treatment. It has reported that RhoA/Rock kinase signaling pathway mediated HSCs activation [34], and RhoA and Rock kinase inhibitors showed anti-fibrosis effect [35]. These findings indicated that ADSC-EXO might suppress HSCs activation by post-transcriptional regulation of key profibrogenic genes.

In the progression of hepatic fibrosis, it was also accompany with activated HSCs proliferation. Next, to ascertain whether ADSC-EXO could inhibit the activated HSCs proliferation, we conducted CCK8 assay to validate LX-2 cell proliferation. As shown in Fig. 4D, the proliferation level of activated LX-2 cells upon ADSC-EXO treatment was apparently decreased than TGF-β primed LX-2 cells. And ADSC-EXO treatment made the proliferation level of activated LX-2 cells be close to untreated LX-2 cells. Consistently, the mRNA level of cell proliferation-related gene RAC1 was profoundly decreased after 24 h ADSC-EXO treatment in activated LX-2 cells (Fig. 4E). Similar to the in vivo results, results of this part indicated that ADSC-EXO showed anti-activation and anti-proliferation ability to HSCs in vitro, which might be a pivotal reason for possessing strong therapeutic potential in hepatic fibrosis.

### Hepatic glutamine synthetase-mediated glutamine and ammonia metabolism was remodeled by ADSC-EXO treatment in hepatic fibrosis mice model

**Fig. 5:**
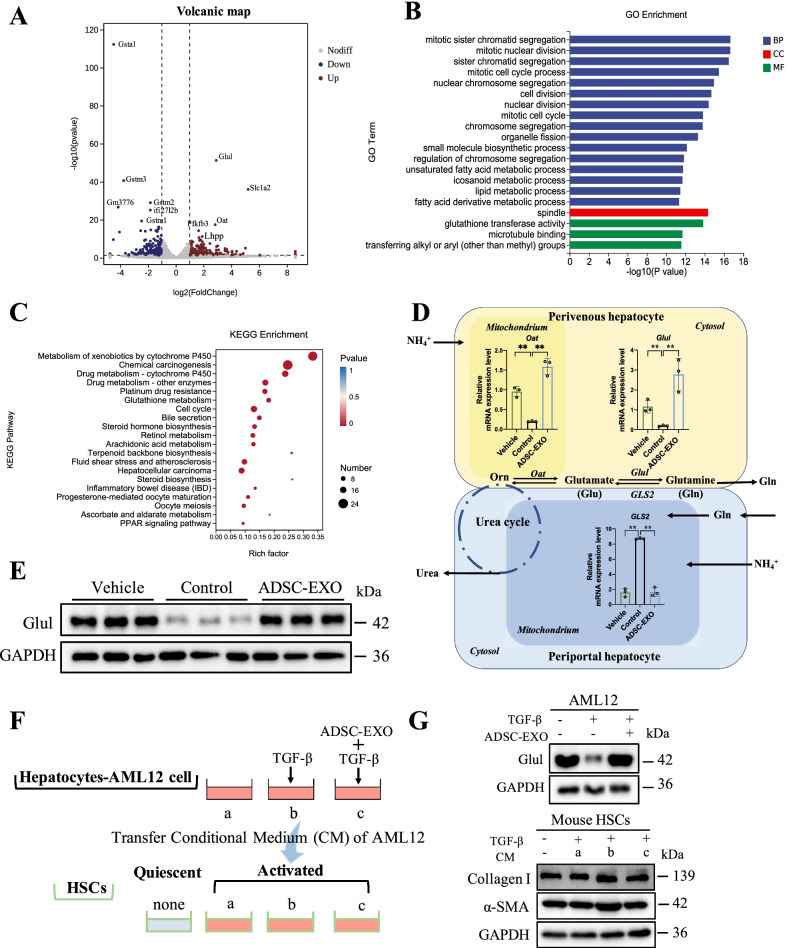
Hepatic Glul was up-regulated in mice treated with ADSC-EXO. RNA-Seq analysis was conducted in total liver mRNA, which was extracted from control mice (DEN/CCl_4_-treated, n = 4) and ADSC-EXO mice liver tissue (DEN/CCl_4_ + ADSC-EXO-treated, n = 4). **A** Volcanic map of different expression genes (DEGs) between control group and ADSC-EXO group. Red spots represent up-regulated genes and blue spots as down-regulated genes. **B** GO term enrichment for control group and ADSC-EXO group. **C** KEGG pathways analysis of control group and ADSC-EXO group. **D** Relative mRNA expression level of key enzymes involving in glutamine and ammonia metabolism in mice liver, including *Glul, GLS2* and *Oat*. Additionally, glutamine and ammonia metabolic process catalyzed by *Glul, GLS2* and *Oat* in perivenous hepatocyte and periportal hepatocyte was described in this diagram. **E** Protein expression level of Glul in mice liver tissue was detected by western blot analysis. (Each lane represents one mouse, 3 replicates.) **F** Scheme illustration of the research model for cross talk between mouse hepatocytes and HSCs. a, b, c all represented the conditional medium (CM) of AML12 cells under three different treatments, respectively. **G** (Up) Western blot analysis of Glul protein level in untreated AML12 cells, AML12 cells with 48 h TGF-β1 treatment (5 ng/ml) for injured and injured AML12 cells exposed to ADSC-EXO (200 μg/ml) for another 24 h. (Down) Western blot analysis of α-SMA and Collagen I protein level in quiescent mHSCs or activated mHSCs with different AML12 cells conditional medium treatment. GAPDH as internal reference. All data are shown as the mean ± SEM, *p < 0.05, **p < 0.01 comparing with corresponding controls by unpaired t test between two groups. Aberrations: DEG, different expression genes; Orn, ornithine; Glu, glutamate; Gln, glutamine; Oat, ornithine δ-aminotransferase; Glul, glutamine synthetase; GLS2, liver-type glutaminase; and CM: conditional medium

To explore the key regulatory factors after ADSC-EXO treatment, we performed RNA-Seq analysis of liver samples in DEN/CCl_4_-treated hepatic fibrosis controls and ADSC-EXO-treated hepatic fibrosis mice. Totally, the expression of 272 genes was significantly up-regulated and 262 genes down-regulated in hepatic fibrosis mice accepting ADSC-EXO therapy. According to the differentially expressed genes, GO enrichment was carried out. The data suggested that cell proliferation-related biological process, cellular component–fatty acid-associated metabolic process and molecular function like glutathione transferase activity were remarkably regulated (Fig. 5B). Besides, KEGG pathway analysis was developed for functional annotation of differentially expressed genes. Of note, KEGG pathway analysis also showed that glutathione metabolism was affected by ADSC-EXO treatment consistently, implying the antioxidant and detoxification role of ADSC-EXO in liver microenvironment (Fig. 5C).

Though the ADSC-EXO treatment exhibited several possible regulations compared with controls, the key beneficial regulator via ADSC-EXO treatment was still uncleared. As depicted in the volcanic map, we found that in up-regulated genes the glutamine synthetase (Glul) transcription level was robustly higher after ADSC-EXO treatment (Fig. 5A). Then, we validated the protein level of Glul in mice liver tissue using western blots. Compared with controls, the results demonstrated that Glul protein level of ADSC-EXO-treated liver fibrosis mice was significant up-regulated (Fig. 5E). As known, glutamine synthetase (Glul) was an essential enzyme in glutamine metabolism, which was responsible for metabolizing glutamate (Glu) into glutamine (Gln) [36] (Fig. 5D). Notably, we also found that another key enzyme in glutamine metabolism, Ornithine δ-aminotransferase, its encoding gene (Oat) transcription level was also higher in ADSC-EXO treatment mice (Fig. 5D). Additionally, mRNA level of liver-type glutaminase (GLS2) in periportal hepatocyte mitochondrium, which was responsible for the reversible reaction of Gln to Glu, was profoundly decreased in ADSC-EXO-treated mice (Fig. 5D). The synchronously rise of Glul and Oat transcription level revealed that ADSC-EXO might mitigate hepatic fibrosis by converting excess ammonia to glutamine, which supports hepatocytes homeostasis and then improves the liver tissue microenvironment.

Within the existing cognitive scope, HSCs activation was the central event in liver fibrosis and there was also cross talk between hepatocytes and HSCs in liver microenvironment. Based on above data, we sought to explore whether ADSC-EXOs contribute to activated HSCs suppression via Glul^+^ hepatocytes. As shown in Fig. 5F, Glul^+^ mouse hepatocytes AML12 cells were treated with TGF-β1 (5 ng/ml) for 48 h to mimic the injured hepatocytes in hepatic fibrosis. In ADSC-EXO treatment group, the injured hepatocytes were following exposed to ADSC-EXOs (200 μg/ml) for another 24 h. Then, we collected and transferred the AML12 cells conditional medium (CM) in each group (a, b, c) to activated mouse HSCs (mHSCs). Firstly, injured AML12 cells showed significantly decrease in Glul protein level, which could be reversed by ADSC-EXO treatment (Fig. 5G). Then, the protein level of α-SMA and Collagen I in mHSCs was examined after 24 h CM treatment. In co-culture of injured AML12 cell CM (b) and activated mHSCs, the protein level of α-SMA and Collagen I in mHSCs was increased than that of mHSCs treated with normal AML12 cells CM (a), while increased expression level of α-SMA and Collagen I was decreased when activated mHSCs treated with ADSC-EXO treatment group CM (c).

### Glutamine synthetase inhibition would erase ADSC-EXO therapeutic effect of hepatic fibrosis

**Fig. 6:**
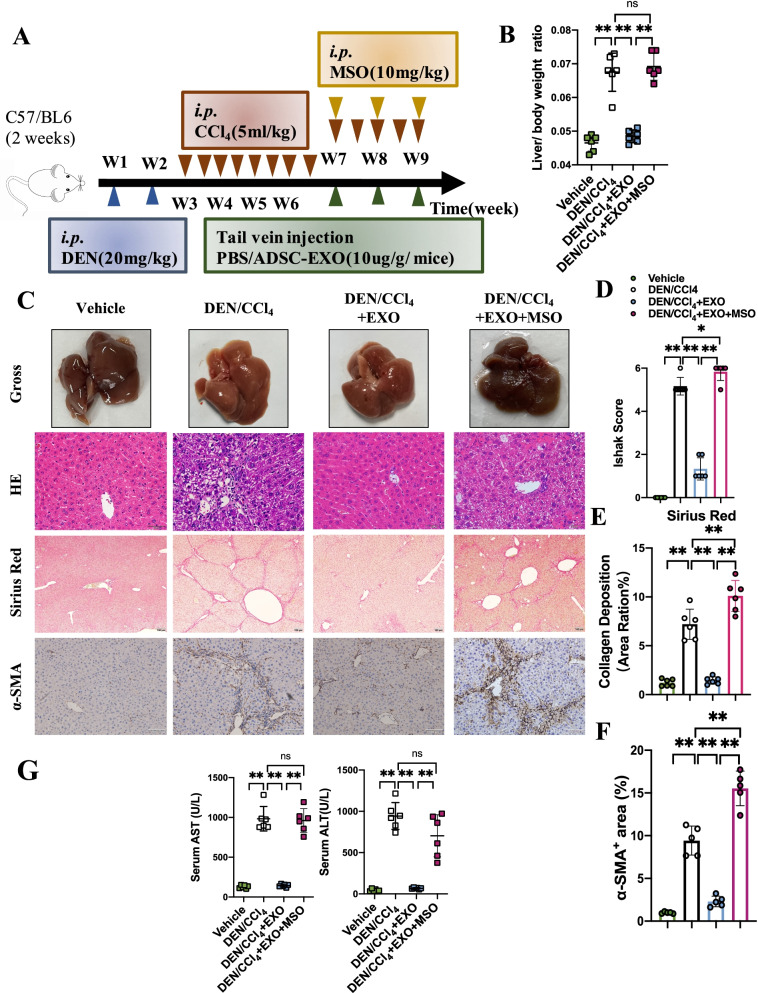

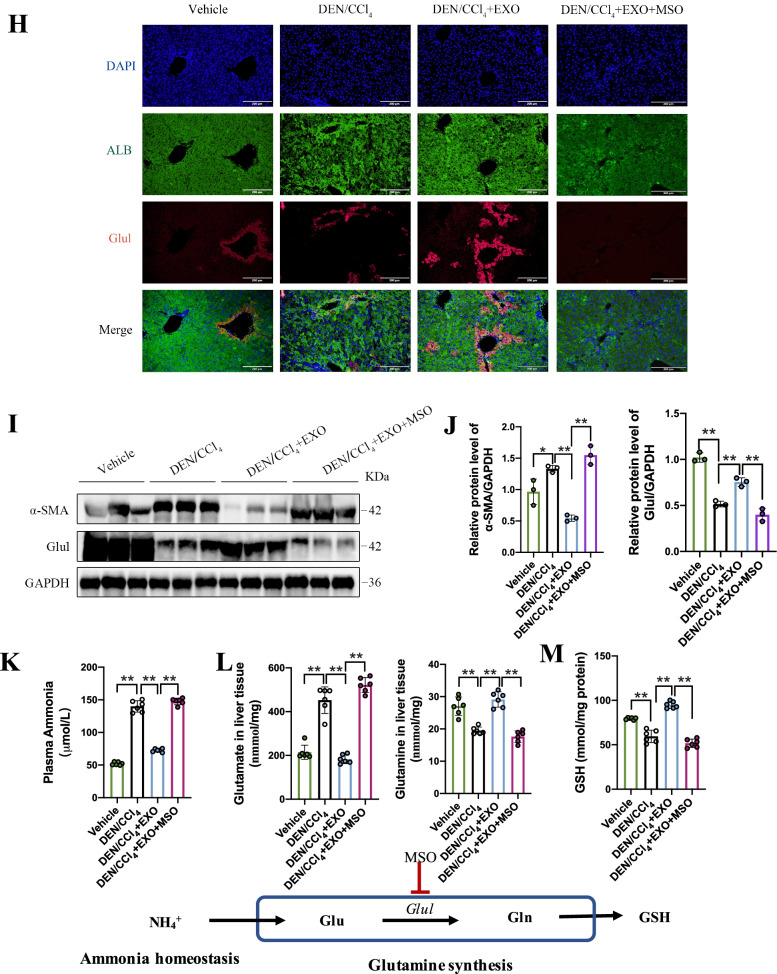
Glutamine synthetase inhibition could erase ADSC-EXO therapeutic effect on hepatic fibrosis. **A** Schematic illustration of DEN/CCl_4_-induced liver fibrosis model, ADSC-EXO treatment strategy and hepatic glutamine synthetase inhibition treated with MSO (10 mg/kg). **B** Liver/body weight ratio of vehicle (PBS), DEN/CCl_4_ mice, DEN/CCl_4_ + EXO mice, DEN/CCl_4_ + EXO + MSO mice (n = 6/group). **C** Representative images of liver gross, H&E staining (scale bar, 50 μm), Sirius Red staining (scale bar, 100 μm) and IHC staining of α-SMA in each group mice liver sections. **D** Ishak score statistical of each group mice. **E** Quantitative statistics of Sirius Red staining by Image J software analysis (6 replicates). **F** Quantification statistic of α-SMA^+^ area% in α-SMA IHC slices by Image J software analysis (5 replicates). **G** Hepatic function serology markers AST and ALT level in mice serum. **H** Representative immunofluorescent staining of ALB (green) and Glul (Red) in each group paraffin-embedded liver sections with different treatments (scale bar, 200 μm). **I** Protein expression level of α-SMA and Glul in mice liver tissue was evaluated by western blot analysis. (Each lane represents one mouse, 3 replicates.) **J** Quantification statistic of (**I**). **K** Plasma ammonia level in each group mice. **L** Glutamate and glutamine concentration in liver tissue. **M** GSH concentration in liver tissue. All data are shown as the mean ± SEM, *p < 0.05, **p < 0.01, comparing with corresponding controls by unpaired t test between two groups. Aberrations: Glu, glutamate; Gln, glutamine; GSH, glutathione

To further analyze the impact of glutamine synthetase on hepatic fibrosis mice, mice were injected methionine sulfoximine (MSO), an inhibitor of Glul, right after ADSC-EXO treatment to deprive the elevation of Glul. Established strategies are shown in Fig. 6A. Liver tissue western blots confirmed that MSO administration after ADSC-EXO treatment significantly reduces the Glul protein level indeed, whereas mice only subject to ADSC-EXO showed high protein level of Glul (Fig. 6I–J). We also confirmed that with the presence of MSO, HSC activation was resurged evidence by elevated α-SMA protein level (Fig. 6I–J). Moreover, IHC staining of α-SMA showed that MSO treatment revived the α-SMA-positive signals than DEN/CCl_4_ + EXO mice (Fig. 6C). Compared with DEN/CCl_4_ + EXO mice, DEN/CCl_4_ + EXO + MSO received mice showed higher liver/body weight ratio, similar to DEN/CCl_4_ mice (Fig. 6B). And serum test showed that MSO increased AST and ALT level, even higher than that in DEN/CCl_4_ hepatic fibrosis controls (Fig. 6G). In corroboration with gross examination, H&E staining and Sirius Red staining results, we found that MSO treatment not only neutralized the ADSC-EXO benefit effect but aggravated liver fibrosis as well, with obvious morphology damage and collagen deposition (Fig. 6C, E–F). Ishak score analysis also demonstrated this result (Fig. 6D), which is represented as significantly elevated scores in DEN/CCl_4_ + EXO + MSO mice. Then, the co-immunofluorescence of hepatocytes marker ALB and Glul showed that Glul expression was expressed limited to 1 layer of perivenous hepatocytes when hepatic fibrosis occurred but Glul expression restored to original level after adopting ADSC-EXO treatment. However, MSO treatment would abrogate the elevated Glul inducing by ADSC exosomes treatment (Fig. 6H).

Glutamine synthesis in perivenous hepatocytes was crucial to scavenge the excess ammonia and supported glutamine metabolism in pathological liver tissue. To investigate the potential effect of up-regulating Glul^+^ perivenous hepatocytes upon treatment with ADSC-EXO, we assessed ammonia homeostasis and glutamine metabolism in mice. ADSC-EXO treatment tended to decrease plasma ammonia levels in DEN/CCl_4_ + EXO group compared with DEN/CCl_4_ group, while this result was not observed in DEN/CCl_4_ + EXO + MSO group (Fig. 6K). Then, glutamate and glutamine concentrations in mice liver tissue were also assessed. Hepatic fibrosis mice treated with ADSC-EXO showed significantly decrease in glutamate level but increase in glutamine level compared with DEN/CCl_4_ group mice, which suggested that the elevated Glul induced by ADSC-EXO treatment enhanced the conversion of glutamate to glutamine (Fig. 6L). Still, MSO treatment would erase the beneficial effect on glutamine homeostasis of ADSC-EXO (Fig. 6L). Due to glutamine as an essential ingredient of glutathione (GSH) synthesis, we next evaluated if the enhanced glutamine flux improved the reduced GSH synthesis in DEN/CCl_4_ group mice liver. Indeed, the GSH level in DEN/CCl_4_ + EXO group mice had increased compared with the DEN/CCl_4_ group mice and DEN/CCl_4_ + EXO + MSO group mice (Fig. 6M).

Taken together, these data provided evidence that elevated Glul induced by ADSC-EXO was of vital importance to liver fibrosis depression, while erasing Glul would exacerbate liver fibrosis.

## Discussion

Based on public health data, chronic liver disease-induced cirrhosis occurs in an estimated 4.5% ~ 9% of all patients with cirrhosis [41]. In the early research stage, liver cirrhosis is considered as an irreversible pathological process, which might eventually develop into HCC, involving in high mortality. However, cirrhosis is a consequence, but not the cause and progression. People should increase the awareness that all severe liver diseases occur on the basis of combination causes, such as virus coinfection, irrational alcohol consumption or chronic hepatitis and so on [42]. After these continuous stimulations, liver would go through a silence and absence of symptoms progressive phase with tissue necroinflammation, then reaching to the stage of fibrosis.

Liver fibrosis is a key but neglected period in the progression of chronic liver disease to liver cirrhosis, which sustains for a relatively long time. Therefore, it provides an optimal treatment window to prevent liver disease progression, reversing means impeding loss of liver function and subsequent liver failure. As a consequence, it is urgent to develop effective and safe strategies to meet the huge clinical need of liver fibrosis treatment. Recent researches reported that varied MSC-derived exosomes exhibited therapeutic effect in tissue damage disease and involved in multiple biological events in paracrine and autocrine way. Of note, these findings bring a conceptual change from cell replacement therapy to cell-free therapy, that is, exosomes involving in immune regulation, tissue repair and microenvironment improving. In this study, we identified that human ADSCs-derived exosomes administration via tail vein could markedly ameliorate DEN/CCl_4_-induced murine liver fibrosis. ADSC-EXO exhibited well performance in suppressing HSCs activation and remodeling hepatocellular glutamine synthetase-mediated glutamine and ammonia metabolism, which might be a promising and multifunctional anti-fibrotic therapeutics for hepatic fibrosis disease.

Actually, exosomes, as a cell-free agent with great potential, are being studied in full swing. In the field of liver disease treatment, more researches emphasize the repair and regeneration role of mesenchymal stem cell exosomes in liver injury. A recent study reported that BMSC-Exos could attenuate hepatocyte apoptosis by promoting its autophagy process in acute liver failure (ALF) [43]. Additionally, studies showed that human umbilical cord mesenchymal stem cells-derived exosomes (hUCMSC-Exo) ameliorated ALF via offering antioxidant hepatoprotection [44] and showing anti-inflammatory effect of hUCMSC-Exo by inhibiting NLRP3 in macrophages [45]. Interestingly, another study concerning adipose mesenchymal stem cell (MSC)-derived exosome (AMSC-Exo) had a similar finding, which AMSC-Exo suppressed NLRP3 inflammasome activation by targeting TXNIP in macrophages [30]. And due to the unique characteristics of exosomes, it is a natural intercellular communication vehicle with abundant functional cargo, which might be delivered to other cells and mediate key cellular process. For example, recent study demonstrated that hUCMSC-Exo cargo BECN1 could induce HSC ferroptosis to achieve hepatic fibrosis mitigation [46]. Wang N *et.al* found that miR-6766-3p in 3D-human embryonic stem cell exosome could target TGFβRII-SMADS pathway and inactivate HSC in liver fibrosis [47]. Notably, from the perspective of biodistribution of EVs, exosomes have great advantages in the treatment of liver diseases. In a systematic review, it summarized the biodistribution of EVs after administration in vivo [48]. It was found that the EVs biodistribution was in a time-dependent manner and liver was the first organ reaching to peak in the first hour and 2–12 h with small-EVs administration. Furthermore, liver was the major organ with the abundant small-EVs. Additionally, different routes of EVs administration also had different impacts on biodistribution pattern. Generally, the most common systemic delivery routes of EVs were intravenous injection (*i.v.*), intraperitoneal injection (*i.p.*) and subcutaneous injection(*s.c.*). It was found that EVs accumulation level in liver tissue with *i.v.* administration was higher than *i.p.* and *s.c.* administration [58]. In more in-depth researches, exosomes might also develop as an ideal drug delivery system. Ma L *et.al* reported that MSC-derived exosomes loaded with therapeutic circRNAs-circDIDO1 suppressed HSC activation in liver fibrosis [49]. In another study, researchers utilized plasma-derived exosomes containing FZD receptor protein (FZD7) to modulate the dysregulated Wnt/Frizzled (FZD) signaling in nonalcoholic fatty liver disease (NAFLD) [50]. These studies unveiled that different kinds of exosomes are natural nanoparticles for rescuing liver injury.

In previous study, we have verified that ADSC-derived extracellular vesicles showed therapeutic effect in ALF model, which improved ALF rat survival rate and recovered liver functions. Accordingly, we speculated that exosomes secreted from ADSC-EXO might show similar therapeutic potential in hepatic fibrosis. To this end, we isolated qualified ADSCs from adipose tissue, then collected and separated ADSC-EVs from Passage 3 to Passage 5 ADSCs. To characterize these EVs, we performed TEM analysis to visualize, NTA analysis to observe its particle size distribution and absolute concentration and western blot analysis to identify its classical protein marker of Alix, Tsg101, CD63 and ER marker Calnexin. These data supported that the we separated ADSC-EXO. In DEN/CCl_4_-induced liver fibrosis mouse model, we preliminary proofed that ADSC-EXO administration for 3 weeks dramatically reversed the fibrotic phenotypes and restored hepatic function compared with control group, including liver morphology recovery, liver/body weight ratio decreased and ALT/AST remission. Notably, one of the specific pathological phenomena to be solved in hepatic fibrosis is the aberrant massive accumulation of collagen. In further analysis, the results of Sirius Red stain indicated that ECM deposition was significantly decreased in these mice treated with ADSC-EXO. Besides, ADSC-EXO treatment also resulted in serum Collagen III and HA reduction, and Collagen I and Collagen VI mRNA level in liver tissue also exhibited the same tendency. Importantly, ADSC-EXO displayed anti-fibrotic effect by down-regulating these profibrogenic genes, such as Acta2, Pdgfr, Tgfb1 and TIMP1.

ECM deposition is usually produced by hyper-activated HSCs, thereby contributing to fibrosis development. The above data had demonstrated that ADSC-EXO could reduce ECM deposition, and thus, we measured HSCs marker α-SMA and Collagen I in mice liver tissue. As expected, western blot analysis and IHC staining results showed decreased expression of α-SMA and Collagen I in vivo. With LX-2 human hepatic stellate cell in vitro model, we confirmed that ADSC-EXO intervention could inactivate the activated LX-2 cells induced by TGF-β. Our results illustrated that ADSC-EXO treatment is capable to relieve HSCs hyper-activation.

Hepatic glutamine synthetase (GLUL) is a critical enzyme in glutamine and ammonia metabolism, which is responsible to synthesis glutamate into glutamine. This process contributes to systematic ammonia detoxification, which maintains corresponding physiological level of ammonia. In liver functional dysregulation disease, glutamine synthesis in perivenous hepatocytes was crucial to scavenge the excess ammonia in pathological liver tissue, maintaining the ammonia metabolic homeostasis. Generally, the main route of excessive ammonia removal in liver mainly relies on two ways. One is urea cycle in periportal hepatocytes, and the other is depending on Glul positive hepatocytes [51]. Unlike urea cycle occurring in mitochondrium of liver-type glutaminase (GLS2) containing periportal hepatocyte as shown in Fig. 5D, Glul-positive cells dominate a cytosol biological process [36]. This cell population in liver was restricted to the very distal portion perivenous hepatocytes, that is, zone 1 of liver. They act as “scavenger” for excessive ammonia that escaped from urea cycle, which provided “the last line of defense” for ammonia homeostasis in organism. In this process, ornithine (Orn) is utilized as a substrate and catalyzed by ornithine δ-aminotransferase (OAT) in mitochondrium, then into glutamate in cytosol [40]. Afterward, under the catalysis of GLUL, glutamate (Glu) serves to form glutamine (Gln) in cytosol of perivenous hepatocytes. Hepatic Glul was primarily expressed in 1–3 layer of perivenous hepatocytes and played role in its cytosol, attributing to glutamine synthesis and having a close relationship with hepatocytes regeneration [37–39]. Notably, compared with urea cycle pathway, Glul-positive hepatocytes-mediated ammonia clearance system exhibits high-affinity but low-capacity character [52].

In early studies, it had found that hepatic glutamine synthetase deficient would trigger systemic hyperammonemia [53], which disturbed ammonia homeostasis maintenance. In other researches, they utilized genetic knockout hepatic glutamine synthetase (LGS-KO) mice as systemic hyperammonemia model, and then, cerebral transcriptome and proteome showed that LGS-KO mice would elevate cerebral oxidative stress level [54]. LGS-KO also had close correlation with hepatic encephalopathy, meanwhile causing irreversible damage of CNS development [55]. But all the above situations occurred when GLUL is genetic deficient. For other liver disease, it was also important to elaborate the Glul change. Firstly, it had observed that hepatic Glul was down-regulated in human liver cirrhosis [56]. Besides, Glul was identified expressing in hepatocyte progenitor cells (HPC) and its normal level associated with hepatocytes regeneration, which loss of expression hint chronic liver disease advanced [37]. Consistently, it had reported that aberrant Glul expression adjacent to portal tracts could be a marker of “regressive fibrosis” that differ from “progressive fibrosis” [57].

In our results, RNA-Seq analysis showed that Glul and OAT were both up-regulated in the liver of ADSC-EXO-treated mice, which was also confirmed by western blots and qRT-PCR. The data suggested that Glul expression of perivenous hepatocytes was down-regulated in hepatic fibrosis mice, while ADSC-EXO treatment could reverse the decreased hepatic Glul expression. Considering the metabolic transversion of glutamate to glutamine, we also evaluated the mRNA level of GLS2 involving in glutamine to glutamate. These results indicated that ADSC-EXO treatment promoted glutamine synthesis reaction through Glul up-regulation and meanwhile suppressed glutaminolysis through GLS2 down-regulation as an auxiliary. Subsequently, to provide a more credible proof that ADSC-EXO induced Glul up-regulation playing therapeutic potential in liver fibrosis, we further used Glul inhibitor MSO to neutralize the enhanced Glul level in ADSC-EXO-treated mice. A series data elucidated that Glul inhibition would erase the ADSC-EXO therapeutic effect on liver fibrosis, which supported that enhanced Glul of perivenous hepatocytes is critically involved in prevention of hepatic fibrosis.

## Conclusions

**Fig. 7:**
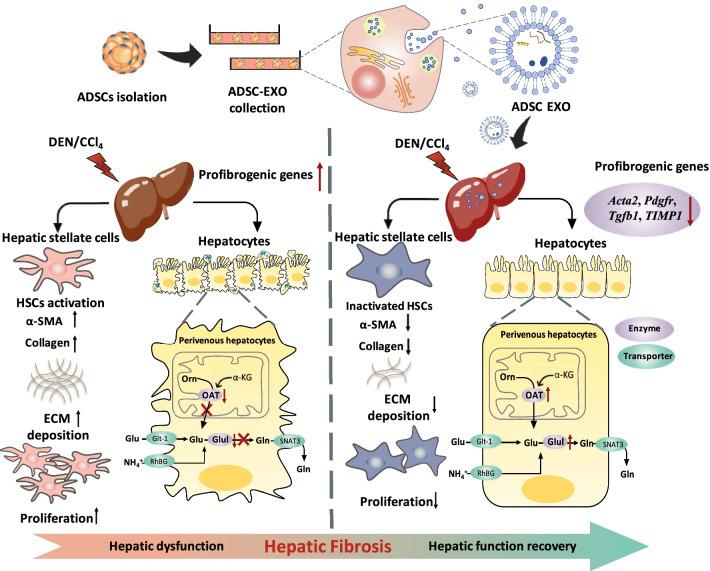
Working model of ADSC-derived exosomes in liver fibrosis. When DEN/CCl_4_-induced liver fibrosis occurred, HSCs cells were activated and highly proliferated, and then, ECM deposition was facilitated. Moreover, glutamine synthetase-positive subset cells of perivenous hepatocytes were dysregulated, which are manifested as two key enzymes for glutamine production OAT and Glul down-regulated, leading to ammonia metabolic homeostasis in liver broken and accelerating hepatic dysfunctions in liver fibrosis. However, our study demonstrated that ADSC-EXO treatment could suppress HSCs activation and weak its proliferation ability, then accompanying with ECM deposition remission. Importantly, ADSC-EXO treatment could restore ammonia metabolic homeostasis by up-regulating OAT and Glul of perivenous hepatocytes. Overall, ADSC-EXO led to hepatic functions recovery in hepatic fibrosis disease

In conclusion, these findings demonstrated the therapeutic effect of ADSCs-derived exosomes on DEN/CCl_4_-induced hepatic fibrosis mice (Fig. 7). We provided evidence that ADSC-EXO possessed the anti-fibrotic potential in hepatic fibrosis, including restoring hepatic functions, promoting liver tissue repairing, reducing ECM deposition, down-regulating these key profibrogenic genes, etc. Importantly, we elucidated that ADSC-EXO treatment could suppress HSCs activation and remodel hepatocellular glutamine synthetase-mediated glutamine and ammonia metabolism, which played a vital role in improving liver microenvironment. Our study supported that ADSC-derived exosomes could be a multifunctional and efficient cell-free therapy against hepatic fibrosis disease.

## Supplementary Information

**Additional file 1**.**Table S1. **Primers Sequences used in q-PCR assay.

## Data Availability

The datasets generated and/or analyzed during the current study are available from the corresponding author on reasonable request.

## Abbreviations

MSCs: Mesenchymal stem cells
ADSCs: Adipose-derived stromal cells
EVs: Extracellular vesicles
ADSC-EXO: ADSCs-derived exosomes
ALF: Acute liver failure
ECM: Excessive extracellular matrix
HSCs: Hepatic stellate cells
α-SMA: α-Smooth muscle actin
TGF-β: Transforming growth factor-beta
ALT: Alanine aminotransferase
AST: Aspartate aminotransferase
COLIII: Collagen III
HA: Hyaluronic acid
LX-2: Human hepatic stellate cell line
Gln: Glutamine
Glu: Glutamate
GLUL (GS): Glutamine synthetase
GLS2: Liver-type glutaminase
OAT: Ornithine δ-aminotransferase
GSH: Glutathione
MSO: Methionine sulfoximine
Orn: Ornithine
Pdgfr: Platelet-derived growth factor receptor
TIMP1: Tissue inhibitor metalloproteinases 3
CV: Central vein
*i.p.*: Intraperitoneal injection
*i.v.*: Intravenous injection
*s.c.*: Subcutaneous injection
GADPH: Glyceraldehyde 3-phosphate dehydrogenase
IHC: Immunohistochemistry
qRT-PCR: Quantitative real-time polymerase chain reaction
IF: Immunofluorescence
CCK8: Cell counting kit-8
RNA-Seq: RNA-sequencing analysis
KEGG analysis: Kyoto encyclopedia of genes and genomes analysis
GO enrichment: Gene ontology enrichment
DEGs: Differentially expressed genes
TEM: Transmission electron microscopy
NTA: Nanoparticle Tracking Analysis
DAB: 3,3′-diaminobenzidine
FBS: Fetal bovine serum
DAPI: 4′,6-diamidino-2-phenylindole
PBS: Phosphate-buffered saline
