# Supplementary material for: ADSCs-derived exosomes ameliorate hepatic fibrosis by suppressing stellate cell activation and remodeling hepatocellular glutamine synthetase-mediated glutamine and ammonia homeostasis
Source: Stem Cell Res Ther. 2022 Oct 4;13:494. doi: 10.1186/s13287-022-03049-x (PMC9531400; doi:10.1186/s13287-022-03049-x)
Supplement: Supplementary file 1 — Additional file 1.Table S1. Primers Sequences used in q-PCR assay. [file 13287_2022_3049_MOESM1_ESM.docx]

**Table S1. Primers Sequences used in q-PCR assay.**

|  | Gene | Forward/  Reverse | Sequences (5’-3’) |
| --- | --- | --- | --- |
| 1 | β-actin (Mus) | Forward | CTTTGCAGCTCCTTCGTTGC |
|  |  | Reverse | CCTTCTGACCCATTCCCACC |
| 2 | Col1a1(Mus) | Forward | ACATGTTCAGCTTTGTGGACC |
|  |  | Reverse | TAGGCCATTGTGTATGCAGC |
| 3 | Col4a4(Mus) | Forward | GTCTGGCTTCTGCTGCTCTT |
|  |  | Reverse | CACATTTTCCACAGCCAGAG |
| 4 | Pdgfr (Mus) | Forward | TTGCCTTACGACTCCACCTG |
|  |  | Reverse | TAGATGGGCCCTCCTTTGGT |
| 5 | Tgfb (Mus) | Forward | ACCGCAACAACGCCATCTAT |
|  |  | Reverse | TGCCGTACAACTCCAGTGAC |
| 6 | TIMP1(Mus) | Forward | GGCATCTGGCATCCTCTTGT |
|  |  | Reverse | TGGTCTCGTTGATTTCTGGGG |
| 7 | Acta2(Mus) | Forward | CCTTCGTGACTACTGCCGAG |
|  |  | Reverse | AATGCCTGGGTACATGGTGG |
| 8 | Oat (Mus) | Forward | GAAATCCCTGGCTCGGTT |
|  |  | Reverse | CCTTGTCTACATCCTTGTTGGT |
| 9 | Glul (Mus) | Forward | GCAAAGACCCCAACAAGCTG |
|  |  | Reverse | GTGGCCGTCTGTTCCCATAA |
| 10 | GLS2 (Mus) | Forward | CTTCCAAAAGTGTGTGAGCAGC |
|  |  | Reverse | GGGATGTAGGCTGCCACTTT |
| 11 | COL1A1 (homo) | Forward | GAACGCGTGTCATCCCTTGT |
|  |  | Reverse | GAACGAGGTAGTCTTTCAGCAACA |
| 12 | TIMP3 (homo) | Forward | ATGCCACCTCCTGAGATCCT |
|  |  | Reverse | AGGATCTCAGGAGGTGGCAT |
| 13 | Fibronectin (homo) | Forward | GCCACTGGAGTCTTTACCACA |
|  |  | Reverse | CCTCGGTGTTGTAAGGTGGA |
| 14 | RHOA (homo) | Forward | GATTGGCGCTTTTGGGTACAT |
|  |  | Reverse | AGCAGCTCTCGTAGCCATTTC |
| 15 | RAC1 (homo) | Forward | ATGTCCGTGCAAAGTGGTATC |
|  |  | Reverse | CTCGGATCGCTTCGTCAAACA |
| 16 | β-actin (homo) | Forward | GGCACTCTTCCAGCCTTCC |
|  |  | Reverse | GAGCCGCCGATCCACAC |
